# When Stress Meets Support: How AI Learning Support Shapes the Link Between Stress Mindset and School Burnout

**DOI:** 10.3390/bs16020220

**Published:** 2026-02-03

**Authors:** Min Ning, Jiaze Lv, Wanying Zhou, Shu Su, Bin-Bin Chen

**Affiliations:** 1 Department of Psychology, Fudan University, Shanghai 200433, China; 2 Wellbeing Research Centre, Harris Manchester College, Oxford University, Oxford OX1 3TD, UK; 3 Department of Early Childhood, Youth, and Family Studies, Ball State University, Muncie, IN 47306, USA

**Keywords:** stress mindset, AI learning support, school burnout, conservation of resources theory, adolescence

## Abstract

School burnout is an increasing concern in highly competitive educational contexts. As artificial intelligence (AI) becomes embedded in classrooms, it shapes both learning processes and students’ stress experiences. Grounded in Mindset Theory and Conservation of Resources framework, this longitudinal study examined whether AI learning support moderates the link between stress mindset and school burnout. A sample of 850 Chinese middle school students (Mage = 15.09, 41% boys) completed two waves of surveys one year apart. Regression results showed that viewing stress as enhancing predicted lower subsequent burnout after controlling for baseline burnout and demographics. Although AI learning support did not directly predict burnout, its interaction with stress mindset was significant: the negative association between a positive stress mindset and burnout was observed when AI learning support was high. These findings suggest that AI can function as an external resource that amplifies adaptive beliefs, offering new pathways for fostering resilience in digital learning environments.

## 1. Introduction

Amid intensifying academic competition, the impact of academic stress on adolescents’ mental health has become a central concern in developmental and educational research. When students lack adequate resources to meet academic demands, they are more vulnerable to emotional exhaustion, disengagement, and social adjustment problems (Bask & Salmela-Aro, 2013; Salmela-Aro & Upadyaya, 2020). Traditionally, investigations of academic stress have emphasized individual traits such as self-efficacy or coping style (Dogan, 2023). Yet, as learning environments become increasingly digitized, artificial intelligence (AI) is shaping not only how students acquire knowledge but also how they experience and manage stress (Yim & Su, 2025). Digitalization intensifies academic pressure by creating an “always-on” learning culture with constant access to assignments and peer comparison (Ding et al., 2025). At the same time, it increases students’ exposure to AI tools that become part of their everyday study routines (Yim & Su, 2025). Understanding the psychological implications of this shift is essential. By examining how AI learning support interacts with students’ appraisals of stress, the present study seeks to illuminate new pathways for protecting adolescents from the escalating problem of school burnout.

### 1.1. School Burnout

Originally conceptualized in occupational settings, burnout refers to a syndrome of emotional exhaustion, depersonalization, or reduced personal accomplishment resulting from chronic stress (Maslach et al., 2001). Subsequent research has established that similar pattern arises in the academic contexts, described as school burnout—a persistent state caused by sustained academic pressure (Salmela-Aro et al., 2009a). Its three dimensions: emotional exhaustion, depersonalization, and reduced personal accomplishment—capture the erosion of motivation and well-being under chronic scholastic demands.

School burnout is widespread and developmentally consequential. It predicts poorer academic achievement (Madigan & Curran, 2021; Salmela-Aro et al., 2009b), higher absenteeism and dropout risk (Bask & Salmela-Aro, 2013), and more depressive symptoms (Fiorilli et al., 2017; Salmela-Aro et al., 2009b). Because adolescence represents a critical window for identity formation and motivational development, chronic burnout during this stage may have enduring effects on psychological adjustment (Tang et al., 2021). Identifying mechanisms that mitigate burnout, therefore, remains a pressing priority for both researchers and educators.

### 1.2. Stress Mindset and School Burnout

Academic stress is one of the most pervasive challenges adolescents faces (Zhou et al., 2023), and a well-established antecedent of school burnout (Teuber et al., 2021; Veyis et al., 2019). However, individuals differ dramatically in how they interpret and respond to stress. Building on Cognitive Appraisal Theory (Folkman et al., 1986), recent research highlights stress mindset—the belief that stress is either enhancing or debilitating—as a key determinant of these responses (Crum et al., 2013). This belief system shapes whether individuals view stress as an opportunity for growth or as a threat to well-being.

According to Mindset theory (Dweck, 2017), adolescents who hold an enhancing stress mindset to view academic pressure as meaningful and manageable, adopt proactive coping strategies, and maintain a stronger sense of purpose and competence (Ryan & Deci, 2000; Zhao et al., 2023). Conversely, those with a debilitating mindset often resort to avoidance, more emotional exhaustion and disengagement. Empirical evidence supports this pattern: a positive stress mindset has been shown to reduce school burnout by strengthening academic resilience (Lakkavaara et al., 2024). Thus, stress mindset functions as an internal psychological resource that may safeguard adolescents against the depletion effects of prolonged academic demands.

### 1.3. The Role of AI Learning Support

Alongside internal resources, technological resources are emerging as powerful contextual support in students’ learning environments. AI-based learning tools, ranging from adaptive tutoring systems (e.g., personalized tutoring platforms like China’s Yuanfudao and Zuoyebang) to generative feedback agents (e.g., ChatGPT or Deepseek), have become integral to daily study routines (Yim & Su, 2025). These tools not only personalize instruction but also promote autonomy, a core element of Self-Regulated Learning theory (SRL) (Deng & Chen, 2025). AI tools facilitate autonomy by supporting goal setting, self-monitoring, and individualized pacing. These features strengthen students’ perceived control and intrinsic motivation when confronting academic challenges (Biju et al., 2024). Moreover, research indicates that AI support can enhance perceived competence, thereby promoting emotional resilience and improving learning performance and capability (Deng & Chen, 2025). Thus, AI learning support is increasingly recognized as a significant external resource.

With the Demands Resources model (Bakker & Demerouti, 2007; Mikolajczak & Roskam, 2018; Salmela-Aro & Upadyaya, 2014; Teuber et al., 2021), burnout emerges when demands chronically outweigh available resources. In this framework, AI-empowered learning tools may function as a novel resource that offsets cognitive and emotional demands by facilitating efficient learning and students’ autonomous engagement (Biju et al., 2024; Song, 2025). Conservation of Resources (COR) theory posits that coping with stress hinges on the dynamic interplay between internal and external resources (Halbesleben et al., 2014). External support amplifies the benefit of internal capacities, whereas resource scarcity weakens them. Specifically, external resources are crucial moderators of how internal resources function. For example, when students perceive higher social support, the positive effect of resilience on academic performance is strengthened, as social support acts as a key “catalyst” by reinforcing the link between resilience and self-regulatory behaviors (K. Li et al., 2024).

Notably, autonomy support—a dimension closely associated with AI learning tools—plays a vital role in how mindsets exert their effects. At the behavioral level, autonomy support enhances the positive impact of growth mindset on academic engagement (Zhang & He, 2025), while at the emotional level, positive autonomy support strengthens emotional resilience and facilitates the role of an adaptive mindset in alleviating anxiety and reducing academic aversion (Johansen et al., 2023; Reeve et al., 2015). Therefore, applying COR theory, we propose that AI learning support enhances the protective role of a positive stress mindset by increasing adolescents’ sense of efficacy and control. Students who perceive greater AI support should experience an enhanced protective effect of positive stress beliefs on school burnout.

### 1.4. The Present Study

Using two-wave longitudinal data from 850 Chinese middle school students, this study investigates how stress mindset and perceived AI learning support jointly shape the development of school burnout over time. The research is situated within China’s highly competitive, exam-oriented educational ecology. This system creates intense, chronic academic demands that, according to the Demands Resources model, significantly heighten the risk of emotional exhaustion and disengagement, leading to a high prevalence of school burnout (S. Guo et al., 2024; D. Guo & Li, 2023). This pressure is profoundly amplified by Chinese cultural tradition that prizes academic achievement, causing competitive intensity to surge markedly beyond the 9-year compulsory education stage. It creates a critical bottleneck at the middle school level, where the high-stakes transition—with only about half of students gaining entry to academic high schools—concentrates immense psychological pressure on adolescents. This pressurized context makes middle school a critical period for intervention.

Meanwhile, within this high-stakes context, digital education initiatives have led to the rapid integration of AI learning tools—such as adaptive tutoring systems and personalized feedback platforms—into classrooms. This proliferation offers an unprecedented opportunity to investigate whether such emerging contextual resources can buffer against burnout. Drawing from Demands Resources theory (Salmela-Aro & Upadyaya, 2014) and the Conservation of Resources framework (Halbesleben et al., 2014), we conceptualize stress mindset as an internal psychological resource and AI learning support as an external technological resource that may interact to reduce burnout. We hypothesize that: (1) A positive stress mindset and higher perceived AI learning support will predict lower subsequent school burnout, and (2) AI learning support will moderate this association, such that the protective effect of a positive stress mindset will be stronger among students reporting high levels of AI learning support.

## 2. Method

### 2.1. Participants

With informed consent from schools, parents, and students, we conducted two waves of online surveys in two prefecture-level cities of moderate socio-economic development—one in Yunnan province and one in Henan province. Henan, a populous central province, represents a context of intense pressure from highly competitive examinations, while Yunnan, a southwestern province with more diverse socioeconomic landscapes, presents challenges related to comparatively limited educational resources. Notably, in both settings, students’ use of AI for learning was not driven by a unified, school-mandated platform but emerged from a ‘bottom-up’, individualized adoption of diverse tools (e.g., generative chatbots like ChatGPT, adaptive learning apps). Selection of these two provinces allows us to capture a spectrum of academic challenges and resource environments relevant to our investigation of school burnout and potential buffering resources. One public middle school was recruited in each city. The first wave (T1) was administered in the spring semester of 2024; the second wave (T2) followed in the spring semester of 2025. Of the initial 1054 students recruited, 850 students remained in the study, yielding a retention rate of approximately 80.6%. The excluded data primarily resulted from incomplete or low-quality responses (e.g., inattentive answering or inconsistent patterns), which were identified through standard data screening procedures. Importantly, there were no significant differences between the initial and retained samples in terms of age, gender, stress mindset, AI learning support, or school burnout. The final sample comprised 850 valid responses. Of these, 348 (40.9%) were boys and 502 (59.1%) girls, with a mean age of 15.09 years (*SD* = 0.75). Ethical approval for the study was obtained from the Institutional Review Board in the School of Social Development and Public Policy at Fudan University (FDU-SSDPP-IRB-2024-1-099).

### 2.2. Measures

#### 2.2.1. Stress Mindset Measure

The Chinese version of the Stress Mindset Measure (Zhao et al., 2025a) was assessed at T1. The scale contains eight items (e.g., “Overall, I believe that stress can promote my personal growth”). Each item is rated on a 5-point scale (0 = completely disagree, 4 = completely agree). The mean score across items serves as the indicator of stress mindset; higher scores reflect a more enhancing mindset toward stress. In the present sample, Cronbach’s α at T1 was 0.70.

#### 2.2.2. School Burnout Inventory

The Chinese version of the School Burnout Inventory (Salmela-Aro et al., 2009a) was administered at both T1 and T2, with a one-year interval. The inventory comprises three dimensions—emotional exhaustion (e.g., “I feel overwhelmed by my schoolwork”), cynicism (e.g., “I often doubt the meaning of studying”), and reduced personal accomplishment (e.g., “I used to have higher expectations of my school performance”)—with a total of nine items rated on a 6-point scale (1 = completely disagree; 6 = completely agree). The mean score across items indicates the level of school burnout; higher scores denote greater burnout. In the present study, Cronbach’s alpha was 0.90 at T1 and 0.95 at T2.

#### 2.2.3. AI Learning Support

In this study, ‘AI Learning Support’ is operationally defined as students’ perceived capability of AI tools to enhance their learning effectiveness and self-directed learning experience. AI Learning Support was assessed at T2. The AI Learning Support questionnaire (B.-B. Chen, 2025) used in this study comprises three items designed to measure the degree of academic support students perceive from AI tools. Items (e.g., “AI enables me to ask deeper or more insightful questions”) were rated on a 5-point scale (1 = strongly disagree; 5 = strongly agree). The development of this scale was primarily grounded in the core constructs of the Technology Acceptance Model (F. Li et al., 2024) and Self-Determination Theory (Ryan & Deci, 2000). Specifically, the items “AI enables me to ask deeper or more insightful questions” and “AI helps me independently learn new knowledge” reflect perceived usefulness (Davis, 1989), indicating that students believe AI can enhance their learning performance. The item “AI helps me explore topics I am genuinely interested in” embodies the support AI provides for learner autonomy and interest exploration (Ryan & Deci, 2000). Collectively, these items cover the key roles of AI as an external learning resource in terms of cognitive support and motivational stimulation, thereby ensuring the content validity of the scale. A confirmatory factor analysis (CFA) confirmed its unidimensional structure. The model demonstrated excellent fit to the data: CFI = 1.00, TLI = 1.00, RMSEA = 0.00, SRMR = 0.00. All factor loadings were statistically significant (*p* < 0.001) and ranged from 0.69 to 0.93, with an average loading of 0.85. These results provide strong evidence for the scale’s structural validity. The AI tools referred to in the survey items include mainstream applications that students are likely to encounter, such as intelligent tutoring systems (e.g., adaptive learning platforms) and generative AI chatbots (e.g., ChatGPT). In this study, Cronbach’s alpha was 0.88.

### 2.3. Data Analysis

The present study employed multiple regression to examine how stress mindset and AI learning support—both independently and interactively—would predict change in school burnout among middle school students. The dependent variable was school burnout measured at T2; the predictors were students’ self-reported stress mindset assessed at T1 and AI learning support assessed at T2. Control variables included child sex and age, and T1 school burnout was entered as a covariate to control prior school burnout levels. Analyses proceeded in two steps. Step 1 tested the main effects of stress mindset and AI learning support on T2 school burnout. After accounting for demographic variables and T1 school burnout, this model evaluated whether stress mindset and AI learning support independently predicted T2 school burnout. Step 2 added the interaction term between stress mindset and AI learning support to determine whether AI learning support moderated the effect of stress mindset on the development of school burnout. The interaction term was the product of the mean-centered AI learning support and stress mindset. To facilitate interpretation, we report only unstandardized regression coefficients (*B*). Standardized estimates were computed solely post-estimation for internal checks by scaling coefficients using variable standard deviations without applying any global standardization to the predictors. If the interaction was significant, the simple slope analyses were conducted at high and low levels (±1 SD) of AI learning support to test the moderation effect. All regressions were performed in R 4.4.1.

## 3. Result

### 3.1. Common-Method Bias Test

Because all study variables were assessed using self-report measures, we examined the potential influence of common method bias. Harman’s single-factor test was conducted on all measurement items through an unrotated exploratory factor analysis. The analysis extracted six factors with eigenvalues greater than 1.0, and the first factor accounted for 30.49% of the total variance—below the commonly accepted threshold of 40% (Malhotra et al., 2006). These results suggest that common method bias was unlikely to substantially affect the observed associations among study variables.

### 3.2. Descriptive Statistics and Bivariate Correlations

Table 1 summarizes the descriptive statistics and zero-order correlations for all study variables. Boys reported a more positive stress mindset and lower school burnout at T1 than girls. Adolescents’ age was negatively associated with T1 stress mindset. T1 stress mindset was positively related to T2 AI learning support and negatively related to school burnout at both time points. AI learning support was negatively correlated with school burnout at T1. Finally, school burnout was highly stable across the two waves.

### 3.3. Effects of Stress Mindset and AI Learning Support on School Burnout

**Figure 1:**
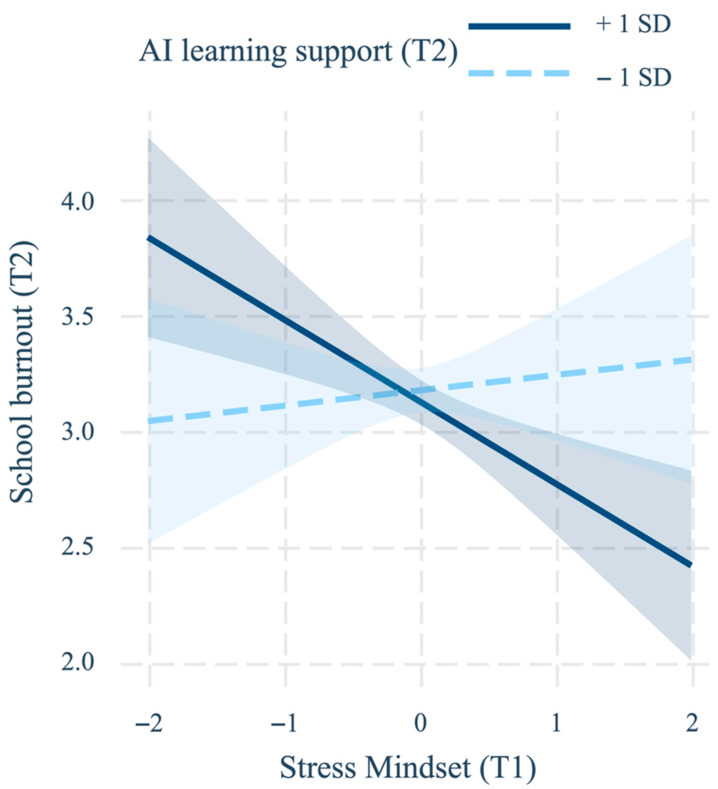
Simple slopes plot of stress mindset when predicting school burnout with AI learning support as moderator. Note. School burnout (T1) and demographic variables were statistically controlled. Values on the *x*-axis represent mean-centered stress mindset (T1) scores. The dashed line depicts the simple slope when AI learning support (T2) is 1 SD below the mean (*B* = 0.066, *p* = 0.618); the solid line represents the simple slope when support is 1 SD above the mean (*B* = −0.354, *p* < 0.001). Shaded bands indicate 95% confidence intervals.

Results of the hierarchical regression analyses are presented in Table 2. Step 1 yielded a significant model, *R*^2^ = 0.185, *F* (5, 844) = 38.42, *p* < 0.001. After controlling for sex, age, and T1 school burnout, T1 stress mindset significantly and negatively predicted T2 school burnout, whereas AI learning support did not predict school burnout. Step 2 introduced the interaction between stress mindset and AI learning support, resulting in a significant increment in explained variance, Δ*R*^2^ = 0.009, Δ*F* (1, 843) = 9.58, *p* = 0.002. The interaction term significantly predicted T2 school burnout (*B* = −0.238, *SE* = 0.091, *p* = 0.002, 95% CI [−0.461, −0.105]), indicating that AI learning support moderated the prospective effect of stress mindset on school burnout; at this stage neither main effect reached statistical significance. Simple slopes analysis (Figure 1) revealed that when AI learning support was low, stress mindset did not predict school burnout (*B* = 0.066, *SE* = 0.133, *p* = 0.618, 95% CI [−0.195, 0.327]). Conversely, when AI learning support was high, a more positive stress mindset significantly predicted lower school burnout (*B* = −0.354, *SE* = 0.104, *p* < 0.001, 95% CI [−0.558, −0.150]). Additional analyses examining the three subdimensions of school burnout yielded highly consistent results and are reported in the Supplementary Materials.

## 4. Discussion

School burnout among adolescents has become a pressing global concern, with competitive educational systems that demand sustained performances. As artificial intelligence (AI) becomes increasingly embedded in classrooms, intelligent systems are reshaping not only how knowledge is delivered but also how students psychologically experience learning. Guided by Demands Resources theory (Salmela-Aro & Upadyaya, 2014) and the Conservation of Resource (COR) framework (Halbesleben et al., 2014), this study explored how AI learning support interacts with middle school students’ stress mindset to influence the development of school burnout. The findings indicate that a positive stress mindset predicts lower school burnout one year later, and that this protective effect is contingent upon perceived AI learning support. Specifically, an adaptive stress mindset buffered burnout only when students reported high levels of AI learning support, suggesting that technological resources can amplify the benefits of positive psychological orientations.

### 4.1. Stress Mindset as an Internal Resource in Preventing School Burnout

According to Dweck’s mindset theory (Dweck, 2017), stress mindset—an individual’s core belief about whether stress is debilitating or enhancing—functions as a cognitive frame that colors both the appraisal of, and coping with, demanding academic events. A more adaptive stress mindset implies richer psychological resources and a greater likelihood of deploying active, problem-focused strategies, thereby interrupting the stress–burnout cycle (Jiang et al., 2025; Lakkavaara et al., 2024). This protective role of positive mindsets against school burnout has been empirically supported across both Western and Eastern cultures (e.g., Altikulaç et al., 2024; Jiang et al., 2025; Lakkavaara et al., 2024). Recent research has further revealed that among Chinese middle school students, a “stress-is-enhancing” mindset is more strongly associated with adaptive learning outcomes (e.g., proactive behaviors), whereas a “stress-is-debilitating” mindset better predicts maladaptive learning outcomes (e.g., academic anxiety, school burnout, and self-handicapping behaviors) (T. T. Chen et al., 2025). Consistent with these findings, the present study also identified the predictive effect of positive mindsets on school burnout among Chinese middle school students, supporting Hypothesis 1. Moreover, after controlling for baseline levels, positive mindsets further predicted lower levels of school burnout one year later, indicating a sustained protective effect and contributing to a deeper understanding of how stress mindsets protect against school burnout. This longitudinal evidence solidly supports the view, derived from Mindset Theory and Cognitive Appraisal Theory, that an “enhancing” stress appraisal serves as a critical internal resource that buffers the impact of chronic academic demands, as framed by the Demands-Resources model.

Furthermore, correlation analyses in this study revealed significant associations between gender/age and stress mindset. Specifically, boys demonstrated more positive mindsets compared to girls, and adolescents in this stage showed declining positivity with increasing age. This finding aligns with conclusions from other studies focusing on middle school students (Zhao et al., 2025b). This pattern may be explained by the emergence of more emotional issues as adolescents progress through this developmental stage, with girls experiencing greater emotional challenges than boys (Bender et al., 2012; Yu et al., 2025), which may subsequently undermine positive mindsets (Ma et al., 2025). Given the stable and sustained protective effect of positive mindsets against school burnout, these results highlight the importance for parents to monitor mindset changes during this developmental period, particularly among female students. By fostering an adaptive stress mindset within a supportive family environment, caregivers can help protect children from burnout while nurturing lasting coping skills that promote both academic success and psychological well-being (Yang & Sun, 2025).

### 4.2. AI Learning Support as an External Resource in Amplifying the Protective Effect of Stress Mindset

The findings highlight a significant interaction between stress mindset and AI learning support. Although AI learning support at T2 was not significantly associated with concurrent burnout levels after controlling for baseline status, the protective association of an adaptive stress mindset was stronger at higher levels of AI learning support. This pattern suggests that internal and external resources function synergistically rather than independently. According to Conservation of Resources theory (Halbesleben et al., 2014) and the Demands–Resources model (Bakker & Demerouti, 2007; Salmela-Aro & Upadyaya, 2014), psychological adaptation hinges on the balances and interplay between personal and environmental resources. Students with an adaptive stress mindset possess a cognitive frame to approach challenges constructively. However, these orientations may only translate into tangible resilience when environmental affordances—such as supportive AI tools—facilitate their implementation.

The regression analysis reveals that AI learning support fails to exhibit a significant direct predictive effect on school burnout. This suggests that, in this sample, AI tools may not function as independent protective resources. Instead, the results are consistent with the idea that they as contextual resources that consistent with the mobilization of students’ existing psychological assets. Consistent with COR theory, external resources exert their strongest influence when they help individuals mobilize internal capacities. AI tools, by enhancing students’ perceived learning effectiveness and supporting autonomous exploration, may correlate with reduced emotional exhaustion primarily when students already hold adaptive stress beliefs. Thus, AI support appears to strengthen resilience pathways indirectly rather than exerting a uniform main effect. It is not a direct ‘remedy’ for alleviating school burnout; rather, it may interact with students’ psychological orientation, such that the protective association of a stress mindset is more pronounced in high-support environments.

In other words, AI learning support acts as a catalytic resource: it does not directly reduce burnout but is associated with students to deploy their internal resources more effectively. The perceived utility of AI in deepening inquiry and facilitating interest-driven learning creates conditions under which adaptive stress mindsets are more likely to be translated into sustained behavioral engagement and improved emotional regulation. In contrast, when such support is lacking, even a positive mindset may fail to protect against chronic stress, as opportunities to enact coping strategies are limited.

Taken together, the proposed model suggests an indirect resource-enactment mechanism: A positive stress mindset provides adolescents with an internal cognitive frame that enables constructive interpretations of academic demands, but this internal resource requires supportive contextual affordances to be translated into adaptive behavior. AI learning tools—by bolstering students’ perception that AI enhances their learning effectiveness and autonomy—enhance their sense of competence and control, thereby allowing an enhancing stress mindset to manifest as effective self-regulation. From the perspective of Self-Determination Theory (Ryan & Deci, 2000), this process can be understood as the AI tools satisfying basic psychological needs for autonomy and competence. This need satisfaction energizes and validates the student’s stress-is-enhancing belief, transforming it from a passive cognition into an active, self-regulated coping process. AI support thus operates not as a direct protective factor but as an environmental affordance that allows stress beliefs to promote sustained engagement and reduced emotional exhaustion.

### 4.3. Practical and Policy Implications

The present findings underscore that the efficacy of internal resources such as stress mindset must be situated within concrete environmental contexts. First, AI learning support may represent not merely as a pedagogical technology but as an external condition associated with the manifestation of positive cognitions into more self-regulated behaviors. Second, in the absence of contextual scaffolding, even an adaptive mindset may be insufficient to counteract chronic academic demands. Consequently, as external support systems, AI learning support may take on greater relevance. Future educational frameworks may conceptualize AI tools as “resource amplifiers”: while cultivating an enhancing stress mindset, they simultaneously supply the affordances (B.-B. Chen, in press)—personalized learning paths that heighten curiosity and perceived control, or just-in-time feedback loops that reinforce persistence—required to translate psychological assets into sustained engagement and reduced burnout. Institutions aiming to bolster student resilience should therefore couple mindset-oriented training with deliberate environmental design. When properly calibrated, AI-infused learning environments can expedite mastery and buffer psychological strain. Policy makers must likewise safeguard educational equity, ensuring that AI-supported resources do not become the privilege of already-advantaged groups, thereby preventing technological dividends from exacerbating systemic inequality.

### 4.4. Contributions and Limitations

Conceptually, the present study frames stress mindset as an internal psychological resource and AI learning support as an external technological resource. Conservation of Resources theory predicts that resource combinations are most protective when diverse assets enhance one another. AI tools can lessen extraneous cognitive load (Biju et al., 2024), expand students’ sense of control, and supply continuous competence feedback—all of which enhance the motivational and emotional benefits of an adaptive stress mindset. From the perspective of Self-Determination Theory (Ryan & Deci, 2000), AI systems that foster autonomy, competence, and relatedness help satisfy basic psychological needs, strengthening intrinsic motivation and promoting well-being. By embedding AI learning support within the COR framework, this study introduces a positive resource-gain perspective, viewing AI not as an isolated utility but as an external resource that synergizes with an adaptive stress mindset to alter the developmental course of school burnout.

The results reported here provide several insights. First, while existing research has largely conceptualized AI in education as an instrumental tool for improving cognitive performance, emotional experiences, or behavioral engagement (Yim & Su, 2025), this study explores how AI-based resources interact with students’ internal psychological assets. Second, by utilizing a two-wave design, we provide preliminary longitudinal evidence of the interplay between stress mindset and modern digital learning contexts. This interactive mechanism offers an empirically grounded, technology-mediated approach to preventing school burnout and enriches the theoretical integration of positive psychology, mindset theory, and the demands–resources model within technology-enhanced educational contexts. Although conducted in China’s high-pressure educational context, the mechanisms identified here are not culture-specific. AI learning tools are now widespread in secondary education globally, and the resource-enactment process we propose—where contextual technological resources amplify internal psychological mindsets—offers a theoretically generalizable account that applies across diverse educational systems.

Despite the aforementioned theoretical and practical contributions, some potential limitations of this study should be acknowledged. First, we measured the study variables with self-report assessment tools. Although the scales demonstrated satisfactory internal consistency in the current sample, future work should triangulate self-reports with behavioral log-files, teacher ratings, or observational measures to enhance validity. Second, although the one-year longitudinal design affords a preliminary test of temporal ordering, the interval is still relatively brief and precludes strong causal inferences. Moreover, the absence of a baseline measure of AI learning support prevents us from examining whether its moderating capacity accumulates, decays, or remains stable over longer periods. Extended longitudinal or experimental micro-intervention studies are needed to clarify causal pathways and to model intra-individual change trajectories. Third, within the Conservation of Resources framework we focused narrowly on the interplay between stress mindset (an internal resource) and AI learning support (an external resource), thereby neglecting other potentially salient assets such as grit, curiosity, academic resilience, or supportive school climate. Integrating a broader resource caravan in future study would illuminate the multifaceted, dynamic transactions that ultimately determine burnout development. Furthermore, while our study conceptualizes AI learning support as a constructive external resource, it is crucial to distinguish this from the potential risk of AI dependency. Excessive or uncritical reliance on generative AI could, paradoxically, undermine the very autonomy and self-regulation it is meant to support (Goh et al., 2025; Huang et al., 2024). This raises a vital question for future research: whether ‘supportive use’ and ‘dependency’ represent distinct—and potentially interacting—pathways that moderate the relationship between stress mindset and burnout. Future studies should examine if the protective effect of AI support observed here is maintained only below certain thresholds of dependency. Fourth, it is noteworthy that the internal consistency reliability of the Stress Mindset Measure in the current study was somewhat lower (Cronbach’s α = 0.70) than the values reported in the previous literature (e.g., Zhao et al., 2025b, αs ≥ 0.76). This discrepancy may be attributable to the unique characteristics of our sample. Our participants were adolescent students in secondary schools, whereas prior studies often utilized samples from general adult populations. It is plausible that the concept of stress mindset is understood or interpreted differently within our specific population, leading to greater variability in responses. Fifth, AI learning support was measured only at T2, preventing us from establishing its temporal precedence as a moderator. Although this raises the possibility that earlier burnout or stress beliefs influenced later perceptions of AI support, COR theory suggests that the contextual resources available at the time still determine whether internal resources can be enacted. Future studies should include baseline and repeated measures of AI support to clarify the direction and stability of this moderating effect. Finally, the sample was drawn from public middle schools in two medium-sized cities in Yunnan and Henan provinces. While this enhances ecological validity relative to single-site studies, cultural and socio-economic homogeneity limits generalizability to rural, high-income, or non-Chinese populations. Cross-cultural and multi-site replication is essential before policy recommendations are exported to markedly different educational ecosystems.

## 5. Conclusions

This study found that, after controlling for baseline levels of school burnout, a stress mindset significantly negatively predicted school burnout one year later. Although the main effect of AI learning support was not significant, it significantly moderated this predictive relationship. Specifically, a significant negative association between stress mindset and school burnout emerged only under conditions of high AI learning support; this protective effect was not significant when support was low.

## Figures and Tables

**Table 1:** Descriptive statistics and correlations among measures.

Variable	*M*	*SD*	1	2	3	4	5	6
1. Gender	0.41	0.49	-					
2. Age	15.09	0.75	0.00	-				
3. Stress mindset (T1)	2.01	0.39	0.11 **	−0.08 *	-			
4. AI learning support (T2)	3.2	0.74	0.05	0.05	0.11 **	-		
5. School burnout (T1)	3.21	1.07	−0.10 **	0.00	−0.39 ***	−0.09 **	-	
6. School burnout (T2)	3.14	1.11	−0.00	0.00	−0.23 ***	−0.07	0.42 ***	-

Note. T1: Timepoint 1; T2: Timepoint 2. * *p* < 0.05. ** *p* < 0.01. *** *p* < 0.001. Gender: 0 = female, 1 = male.

**Table 2:** Prediction of school burnout.

	Step 1			Step 2		
	*B*	*SE*	95% CI	*B*	*SE*	95% CI
Gender	0.108	0.070	[−0.029, 0.245]	0.099	0.070	[−0.038, 0.236]
Age	−0.005	0.046	[−0.095, 0.085]	−0.006	0.046	[−0.096, 0.086]
School burnout (T1)	0.405 ***	0.035	[0.336, 0.474]	0.408 ***	0.035	[0.339, 0.477]
Stress mindset (T1)	−0.223 *	0.095	[−0.409, 0.037]	−0.144	0.098	[−0.336, 0.048]
AI learning support (T2)	−0.038	0.047	[−0.130, 0.054]	−0.036	0.046	[−0.126, 0.054]
Stress mindset (T1) × AI learning support (T2)				−0.283 **	0.091	[−0.461, 0.105]
*R* ^2^	0.185			0.195		
*F*	*F* (5, 844) = 38.42 ***			*F* (6, 843) = 33.94 ***		
Δ*R*^2^	0.009 **					

Note. T1: Timepoint 1; T2: Timepoint 2. * *p* < 0.05. ** *p* < 0.01. *** *p* < 0.001. Gender: 0 = female, 1 = male.

## Data Availability

The original contributions presented in this study are included in the article. Further inquiries can be directed to the corresponding author.

## Supplementary Materials

The following supporting information can be downloaded at https://www.mdpi.com/article/10.3390/bs16020220/s1, Table S1: Prediction of emotional exhaustion; Table S2: Prediction of cynicism; Table S3: Prediction of reduced accomplishment.
