# Supplementary material for: When Stress Meets Support: How AI Learning Support Shapes the Link Between Stress Mindset and School Burnout
Source: Behav Sci (Basel). 2026 Feb 3;16(2):220. doi: 10.3390/bs16020220 (PMC12938416; doi:10.3390/bs16020220)
Supplement: Supplementary file 1 [file behavsci-16-00220-s001.zip › behavsci-4079363-supplementary.pdf]

Supplementary Materials for

When Stress Meets Support: How AI Learning Support Shapes the Link Between Stress Mindset and School Burnout

Min Ning 1, Jiaze Lv 1,Wanying Zhou 2, Shu Su 3 and Bin-Bin Chen 4, *

1 Fudan University
2 Oxford University
3 Ball State University
4 Fudan University
*   Correspondence: chenbinbin@fudan.edu.cn

Table S1.

Prediction of emotional exhaustion.

	Step 1			Step 2		
	B	*SE*	95% CI	*B*	*SE*	95% CI
Gender	0.036	0.073	[-0.107, 0.179]	0.032	0.073	[-0.111, 0.175]
Age	0.012	0.048	[-0.082, 0.106]	0.011	0.047	[-0.081, 0.103]
Emotional exhaustion (T1)	**0.352*****	0.035	[0.283, 0.421]	**0.356*****	0.035	[0.287, 0.425]
Stress mindset (T1)	**-0.091****	0.098	[-0.283, 0.101]	-0.062	0.101	[-0.260, 0.136]
AI learning support (T2)	-0.012	0.048	[-0.106, 0.082]	-0.010	0.048	[-0.104, 0.084]
Stress mindset (T1) × AI learning support (T2)				**-0.104****	0.094	[-0.288, 0.080]
R^2	0.154			0.164		
F	*F* (5, 844) = 30.71***			*F* (6, 843) = 27.53***		
ΔR^2	0.010**					

Note. T1: Timepoint 1; T2: Timepoint 2. *p < .05. ** p < .01. *** p < .001. Gender: 0 = female, 1= male.

Table S2.
Prediction of cynicism.

	Step 1			Step 2		
	B	*SE*	95% CI	*B*	*SE*	95% CI
Gender	0.064	0.076	[-0.085, 0.213]	0.060	0.075	[-0.087, 0.207]
Age	-0.013	0.050	[-0.111, 0.085]	-0.014	0.049	[-0.110, 0.082]
Cynicism (T1)	**0.385*****	0.032	[0.322, 0.448]	**0.389*****	0.032	[0.326, 0.452]
Stress mindset (T1)	**-0.091****	0.102	[-0.291, 0.109]	-0.061	0.105	[-0.336, 0.145]
AI learning support (T2)	-0.027	0.050	[-0.125, 0.071]	-0.026	0.050	[-0.124, 0.072]
Stress mindset (T1) × AI learning support (T2)				**-0.104****	0.098	[-0.296, 0.088]
R^2	0.185			0.195		
F	F (5, 844) = 38.21***			F (6, 843) = 33.96***		
ΔR^2			0.010**			

Note. T1: Timepoint 1; T2: Timepoint 2. *p < .05. ** p < .01. *** p < .001. Gender: 0 = female, 1= male.

Table S3.

Prediction of reduced accomplishment.

	Step 1			Step 2		
	B	*SE*	95% CI	*B*	*SE*	95% CI
Gender	0.059	0.078	[-0.094, 0.212]	0.053	0.078	[-0.100, 0.206]
Age	-0.034	0.051	[-0.134, 0.066]	-0.035	0.051	[-0.135, 0.065]
Reduced accomplishment (T1)	**0.303*** **	0.031	[0.242, 0.364]	**0.301*** **	0.031	[0.240, 0.362]
Stress mindset (T1)	**-0.317** **	0.103	[-0.519, 0.115]	**-0.268* **	0.106	[-0.476, -0.060]
AI learning support (T2)	-0.083	0.052	[-0.185, 0.019]	-0.082	0.051	[-0.182, 0.018]
Stress mindset (T1) × AI learning support (T2)				**-0.189* **	0.101	[-0.387, 0.009]
R^2	0.142			0.146		
F	F (5, 844) = 27.95***			F (6, 843) = 23.94***		
ΔR^2			0.004**			

Note. T1: Timepoint 1; T2: Timepoint 2. *p < .05. ** p < .01. *** p < .001. Gender: 0 = female, 1= male.
